# An X-ray diffractometer using mirage diffraction

**DOI:** 10.1107/S1600576714012114

**Published:** 2014-07-19

**Authors:** Tomoe Fukamachi, Sukswat Jongsukswat, Dongying Ju, Riichirou Negishi, Keiichi Hirano, Takaaki Kawamura

**Affiliations:** a Saitama Institute of Technology, Fukaya, Saitama 369-0293, Japan; b Institute of Material Structure Science, KEK-PF, High Energy Accelerator Research Organization, Tsukuba, Ibaraki 305-0801, Japan; c University of Yamanashi, Kofu, 400-8510, Japan

**Keywords:** mirage diffraction, mirage fringes, interference fringes, X-ray difractometers, monochromators, dynamical theory of X-ray diffraction

## Abstract

Some characteristics are reported of a triple-crystal diffractometer with a (+, −, +) setting of Si(220) using mirage diffraction. The first crystal is flat, while the second and third crystals are bent. Basically, the first crystal is used as a collimator, the second as a monochromator and the third as the sample. The third crystal also works as an analyzer. The advantages of this diffractometer are that its setup is easy, its structure is simple, the divergence angle from the second crystal is small and the energy resolution of the third crystal is high, of the order of sub-meV.

## Abstract 2

Some characteristics are reported of a triple-crystal diffractometer with a (+, −, +) setting of Si(220) using mirage diffraction. The advantages of this diffractometer are that its setup is easy, its structure is simple, the divergence angle from the second crystal is small and the energy resolution of the third crystal is very high.

## 1. Introduction   

The refracted beam of an X-ray in a bent perfect crystal propagates along a hyperbolic trajectory and comes back to the incident surface in the Bragg geometry. The refracted beam is referred to a beam representing the Poynting vector of the X-ray in this paper. Authier (2001 ▶, p. 355) pointed out that the behavior of the refracted beam in a bent crystal resembled a mirage in optics. We call the diffracted beam coming out of the crystal a mirage diffraction beam. Under anomalous transmission conditions, the divergence angle of the refracted beam is quite large compared with that of the incident beam. The refracted beam can be regarded as a quasi-spherical wave, even when the divergence angle of the incident beam is smaller than 1′′ (Authier, 2001 ▶, p. 313). When mirage diffraction beams interfere with each other, this results in interference fringes, which are called mirage interference fringes (Fukamachi *et al.*, 2010 ▶). Such mirage interference fringes were first observed by Zaumseil (1978 ▶). Mirage interference fringes have been used for evaluation of the strain gradient in a bent crystal (Jongsukswat *et al.*, 2012 ▶).

In this paper, we will describe a triple-crystal diffractometer using mirage interference fringes of Si(220) and some characteristics of the diffractometer.

## 2. Theoretical basis   

**Figure 1:**
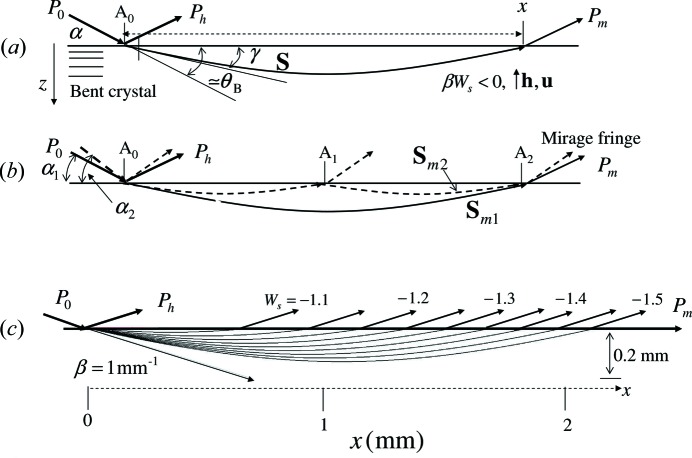
Schematic illustrations of beam geometries in a bent single crystal. (*a*) The trajectory of the refracted beam in a bent crystal. (*b*) Trajectories of mirage diffraction beams emitted from *A*_2_, showing interference fringes. (*c*) Trajectories of the refracted beams for 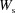 from −1.1 to −1.5, when the X-ray energy is 11 100 eV and 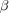 is 1.0 mm^−1^ for the Si(220) reflection.

According to Gronkowski & Malgrange (1984 ▶), the trajectory of the refracted beam in a bent crystal is given for the symmetric Bragg geometry as
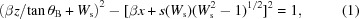
for 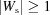, where 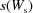 = −1 for 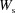 < −1 and 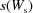 = 1 for 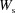 > 1. 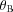 is the Bragg angle, and *x* and *z* are the coordinates parallel and normal to the crystal surface in the incident plane, respectively, with the origin at the incident point of the X-ray on the surface. The parameter 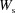 is the initial value of the deviation from the Bragg condition *W*, which is defined by
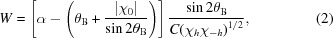
with 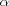 being the incident glancing angle and *C* the polarization factor. 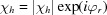 is the *h*th Fourier coefficient of the X-ray polarizability and 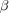 is a parameter corresponding to the strain gradient in the crystal, defined by
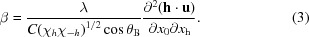
Here, 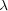 is the X-ray wavelength, **h** the reciprocal vector corresponding to the **h**th reflection and 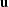 the displacement vector; 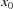 and 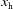 are the coordinates in the directions of the transmitted and diffracted beams, respectively. Under anomalous transmission conditions (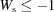), the trajectory of the refracted beam has a hyperbolic form, as shown in Fig. 1 ▶ for 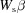 < 0. The eccentricity of the hyperbola is related to 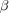. By taking derivatives of equation (2), the divergence angle (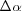) of the incident beam is related to the change of *W* as
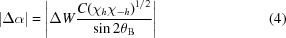
when 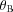 is fixed. In Fig. 1 ▶(*a*), the angle 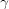 between the refracted beam and the surface is given by
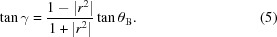
Here the reflectivity (*r*) is defined by 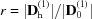 with 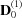 and 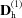 being the electric displacement vectors of the incident and diffracted beams, respectively. The superscript on 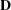 represents the branch index. For a non-absorbing crystal, *r* is expressed by
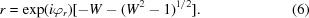
For *W* = −1, 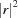 = 1 and 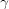 = 0, and for *W* = −2, 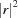 = 0.07 and 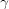 is approximately equal to 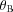. The angle amplification rate *A* between the changes in 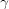 and 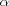 in reflection geometry is given by Authier (2001 ▶, p. 313) as
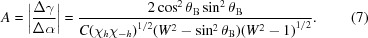
Since *A* is between 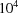 and 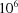, even when 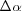 is less than 1′′ and the incident beam can be regarded as a quasi-plane wave, 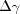 is nearly equal to the Bragg angle, and the refracted beam can be regarded as a quasi-spherical wave if 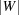 is close to 1. This means that this single crystal works as a lens. This angle amplification can also be applied to a monochromator reflecting X-rays with very small divergence angle as well as to an analyzer with high energy resolution. Authier (1960 ▶) obtained a highly collimated incident beam by using this angle amplification in the transmission geometry so as to verify the double refraction.

In Fig. 1 ▶(*b*) is shown a schematic illustration of interference fringes between two mirage diffraction beams. The refracted beams 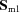 and 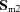 correspond to the incident beams for the values of 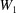 and 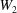, respectively, satisfying the relation 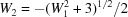. In Fig. 1 ▶(*c*), trajectories are shown of the refracted beams when 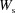 changes from −1.1 to −1.5 in the case of the Si(220) reflection. The X-ray energy is 11 100 eV and 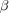 is 1 mm^−1^.

The intensities of the mirage diffraction beams are measured as a function of the distance *x* from the incident point of the beam to the emission point of the mirage diffraction beam. By using equation (1), the deviation (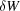) of the parameter of 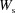 from −1 is given by
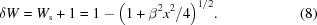



**Figure 2:**
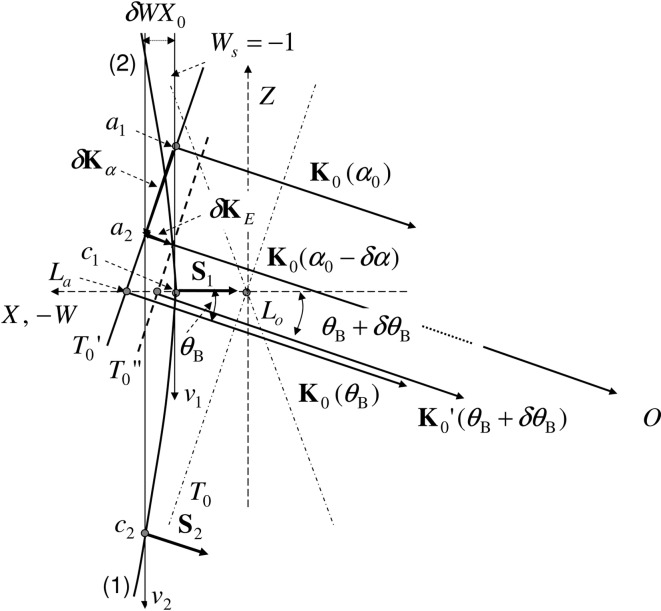
Dispersion surface in a Bragg geometry. *L*_a_ is the Laue point, *L*_o_ the Lorentz point and *c*_1_ the tie point for *W*_s_ = −1. The solid line 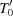 represents the dispersion surface in a vacuum for an incident beam of energy *E* and the solid curve the corresponding dispersion surface of branches (1) and (2) in the crystal. The dot–dashed curve 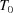 represents the asymptote of the dispersion surface in the crystal. The dashed line represents the dispersion surface in a vacuum for an X-ray of energy 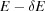. The angle change 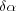 gives the same change in 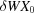 as the change in the wavevector 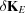 gives when the energy changes from *E* to 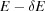. The relations 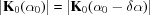 and 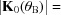 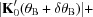 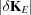 hold.

It is possible to get the value of 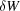 by measuring the position of mirage interference fringes *x*. Fig. 2 ▶ shows the diffraction geometry for an X-ray with energy *E* and glancing angle 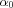. The thick solid line shows the dispersion surface in the crystal for the Bragg angle 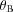 in the Bragg arrangement. *L*_o_ is the Lorentz point, *L*_a_ is the Laue point and *c*_1_ on the *X* axis is the point corresponding to *W*_s_ = −1. The nomenclature of the points *L*_o_ and *L*_a_ is adopted according to the books by Pinsker (1977 ▶) and Authier (1960 ▶, pp. 68–71). The line *T*_0_′ represents the dispersion surface in a vacuum and is parallel to the asymptote *T*_0_ of the hyperbola of the dispersion surface in the crystal. 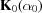 is the wavevector of the incident beam whose glancing angle is 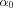 and energy is *E*. We assume that the perpendicular line *v*_1_ passing the tie point corresponding to 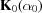 crosses the dispersion surface at *c*_1_. The refracted beam 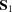 excited at the point *c*_1_ runs parallel to the crystal surface. The distance 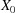 from *L*_o_ to *c*_1_ is given by
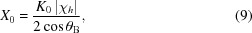
where 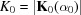, *C* = 1 for 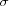 polarization and 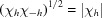. When the glancing angle changes from 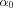 to 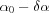, the corresponding perpendicular line changes from *v*_1_ to *v*_2_ and the parameter 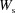 changes from −1 to 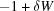. The tie point in a vacuum moves from *a*_1_ to *a*_2_ and that in the crystal moves from *c*_1_ to *c*_2_. From the tie point *c*_2_, the refracted beam 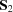 is excited. In Fig. 2 ▶, the relation
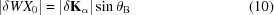
is obtained.

## 3. Experimental   

**Figure 3:**
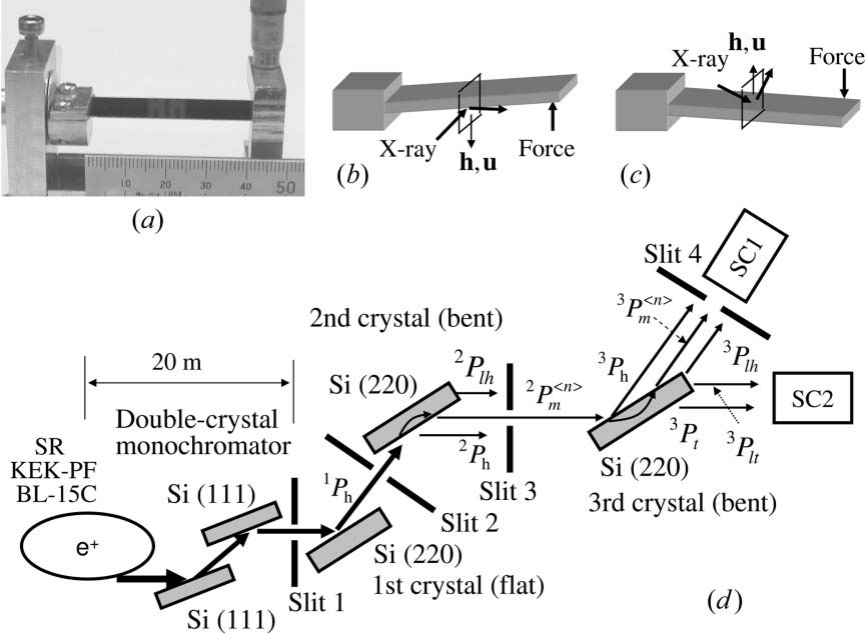
(*a*) Photograph of the cantilever jig and the crystal. (*b*) Geometry of the second crystal. The force is applied in the opposite direction to gravity. (*c*) Geometry of the third crystal. The force is applied in the direction of gravity. (*d*) Schematic diagram of the triple-crystal diffractometer with (+, −, +) setting. The vertical width of Slit 2 is 0.02 mm, and those of Slit 3 and Slit 4 are 0.04 mm.

The experiment was carried out by using X-rays from synchrotron radiation at BL-15C, KEK-PF, Tsukuba, Japan. The optical system is shown in Fig. 3 ▶(*d*). The X-rays were 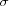 polarized and the energy was tuned to 11 100 ± 0.5 eV by using a double-crystal Si(111) monochromator. After Slit 1, the first crystal was basically used as a collimator, the second as a monochromator and the third as the sample, as shown in Fig. 3 ▶(*d*). The first crystal was flat. The second and third crystals were bent by applying force in the backward and forward directions of gravity, as shown in Figs. 3 ▶(*b*) and 3 ▶(*c*), respectively. The three plane parallel crystals were prepared by non-disturbance polishing at Sharan Instrument Corporation. The crystals were 50 mm long, 15 mm wide and 0.28 mm thick. The usual Bragg diffracted beam (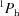) passed through the second slit (Slit 2), and the first peak of the mirage interference fringes (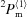) passed through the third slit (Slit 3). Here the left superscript on 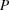 represents either the first (1), second (2) or third (3) crystal. The right superscript on 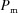 represents a serial peak number of the mirage interference fringes.

**Figure 4:**
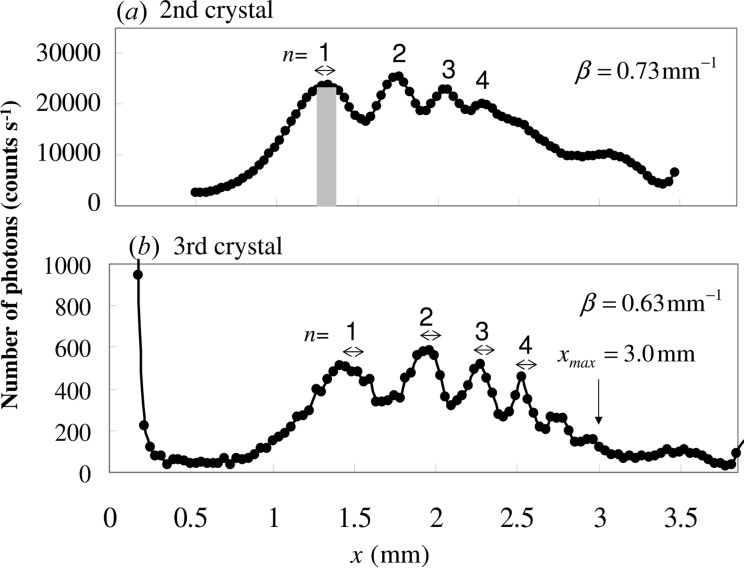
Intensities of mirage interference fringes as a function of the distance *x*. (*a*) Intensities of mirage interference fringes from the second crystal and (*b*) those from the third crystal.

The mirage diffraction intensities of 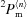 from the second crystal are shown as a function of the distance *x* in Fig. 4 ▶(*a*), where the glancing angle was fixed and β was 0.73 mm^−1^. The intensities were measured by moving Slit 3 in front of the scintillation counter SC2 in Fig. 3 ▶(*d*) after removing the third crystal. The value of β was determined by measuring the position of the third peak as reported by Jongsukswat *et al.* (2012 ▶). The mirage diffraction intensities of 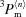 from the third crystal are shown as a function of *x* in Fig. 4 ▶(*b*), where the glancing angle was fixed and β was 0.63 mm^−1^. The intensities were measured by moving Slit 4 in front of the scintillation counter SC1. The vertical width of Slit 2 was equal to 0.02 mm and those of Slit 3 and Slit 4 were 0.04 mm.

**Figure 5:**
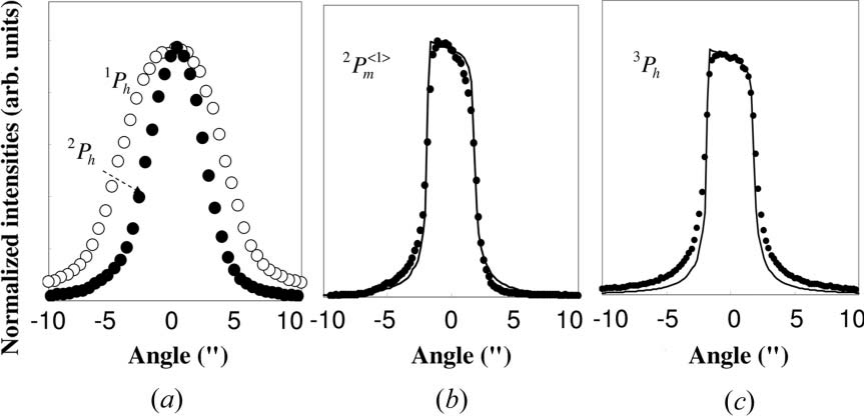
(*a*) Rocking curve of 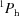 from the first crystal (open circles) and that of 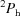 from the second crystal (solid circles). (*b*) Rocking curve of 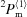 from the second crystal (solid circles). (*c*) Rocking curve of 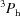 from the third crystal (solid circles). The solid curves in (*b*) and (*c*) are the curves of 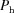 for the Si(220) reflection calculated by taking the absorption effect into account.

**Figure 6:**
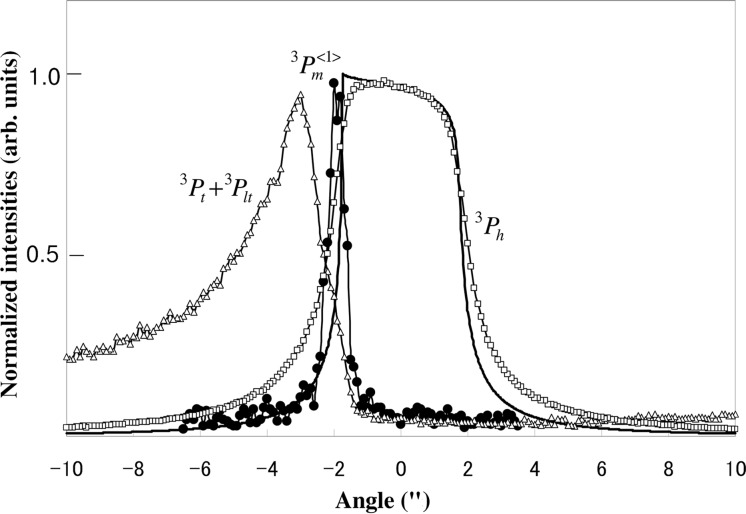
Rocking curves of 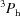 (open circles), 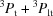 (triangles) and 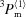 (solid circles) from the third crystal. The solid line is the calculated curve of 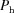.

Rocking curves of 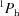 from the first crystal and 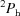 from the second crystal are shown in Fig. 5 ▶(*a*). The FWHM of the curve 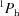 is 9′′, which is twice as large as that of the curve 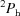 of 4.5′′. The rocking curve of the first peak (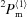) of the mirage interference fringes, with an FWHM of 3.8′′, is shown in Fig. 5 ▶(*b*) (dots). It has an asymmetric form characteristic of the rocking curve from a weakly absorbing crystal. The slopes of both shoulders of the peak are steeper than those of 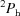. The rocking curve of 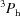 from the third crystal is shown in Fig. 5 ▶(*c*) (dots). It also has an asymmetric form and is in good agreement with the curve (solid line) calculated by taking the absorption effect into account. The FWHM of the peak is 4′′. The rocking curves of 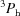, 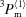 and 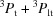 from the third crystal are shown in Fig. 6 ▶. The ordinate is the intensities and the abscissa is the incident angle. The origin of the angle (0) is taken at the center of 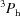, which corresponds to the Lorentz point. The mirage fringe intensity 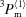 is measured by the scintillation counter SC1 after setting Slit 4 at the peak position of the curve of 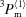. 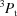 is the intensity of the transmitted beam and 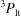 is the intensity of the emitted beam in the direction of the transmitted beam from the lateral surface of the crystal. These intensities are measured by the scintillation counter SC2. The sharp peak of 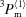, with an FWHM of 0.6′′, appears between the peaks of 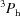 and 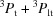. The peaks of 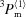 and 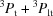 appear on the negative angle side where the anomalous transmission occurs.

**Figure 7:**
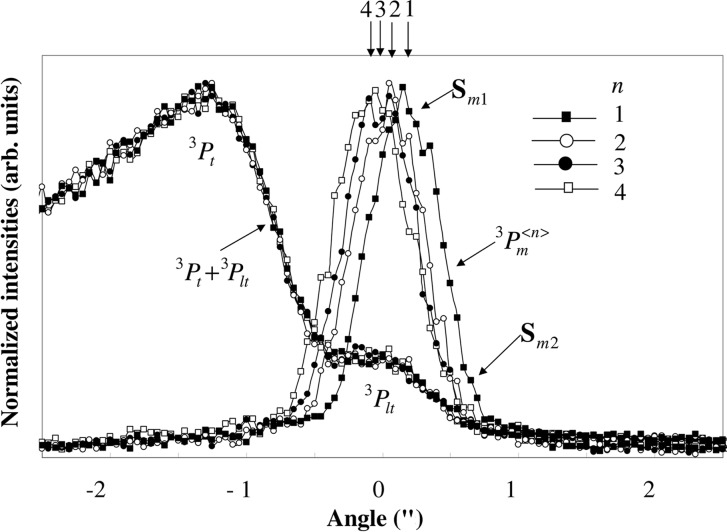
Rocking curves of 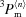 and 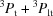 from the third crystal. Solid squares shows 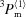, open circles 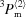, solid circles 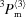 and open squares 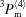. The rocking curves of 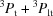 (left) were measured simultaneously with the curve of 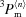 with *n* = 1, 2, 3 and 4. The peak angles of 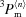 for *n* = 1, 2, 3 and 4 correspond to the angles of the refracted beam 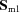 in Fig. 1 ▶(*b*). The shoulder structures of 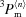 on the high-angle side are probably caused by the component of the refracted beam 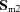

In Fig. 7 ▶ are shown the four rocking curves 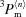 for *n* = 1, 2, 3 and 4 from the third crystal, together with the rocking curves of 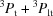. The origin of the angle is taken at the center of the peak of 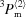. Each curve of 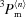 was measured by the scintillation counter SC1 after setting Slit 4 at the peak position of the mirage interference fringes, and the curve of 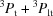 was measured simultaneously by the scintillation counter SC2, as shown in Fig. 3 ▶(*d*). When *n* increases, the peak position of 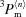 moves to the lower incident angle side, close to the peak of the curve 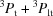. The angle difference between the two peaks 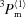 and 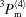 is 0.3′′. The average angle difference between two adjacent peaks is 0.1′′. The curve of 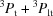 shows two peaks: one, corresponding to the peak of 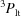, appears around the origin, and the other, corresponding to the peak of the transmitted beam 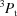, appears around the angle of −1.3′′. When the rocking curves of the *n*th (*n* = 1, 2, 3 and 4) peak of the interference fringes 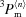 are measured after setting Slit 4 at its peak position, the measured peak of 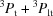 stays at the same position as shown in Fig. 7 ▶. The peak of 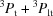 is a good reference point for the incident angle. It is possible to determine a very small angle difference between two peaks of 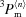 and 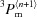 by measuring the curves of 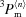 and 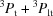 simultaneously.

## 4. Discussion   

Equation (1) derived by Gronkowski & Malgrange (1984 ▶) is applicable only to a monochromatic X-ray of a plane wave. In the present experiment, however, since the X-rays from synchrotron radiation are emitted from a source of finite size and are monochromated by a crystal monochromator, they have a small energy bandwidth as well as a small divergence angle. It is noted that 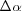 denotes the divergence angle for monochromatic X-rays and 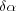 denotes the angle shift corresponding to the energy shift 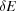, which can be estimated from the observed range of mirage interference fringes. 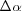 is equal to the maximum value of 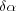. It is necessary to have the relation between the diffraction of monochromatic X-rays and that of X-rays with a finite energy bandwidth and a finite divergence angle. If the X-ray energy changes from *E* to 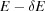 and the amplitude of the wavevector changes from 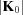 to 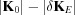 while the glancing angle is fixed, the dispersion surface *T*_0_′ moves to *T*_0_′′ and the Bragg angle changes from 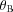 to 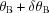 as shown in Fig. 2 ▶. Here 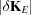 is the amplitude of the wavevector 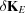, which is related to the energy deviation 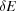 by
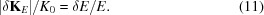
In Fig. 2 ▶, we have the relation between 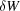 and 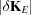 as
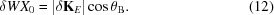
Using equations (9), (11) and (12), 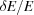 is expressed by
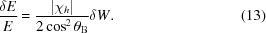
When the dispersion angle changes from 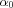 to 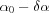, the relation 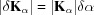 holds in Fig. 2 ▶. 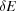 is related to 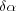 as
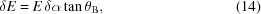
by using the relation 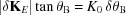 or 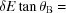 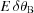.

By inserting equation (13) into equation (8), the energy shift 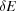 is related to the distance *x* by
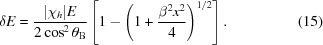
It is possible to estimate 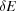 by measuring *x*. In the present experiment, since the measured maximum value of *x* is 3 mm, as shown in Fig. 4 ▶(*b*), the maximum value of 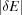 is obtained as 11 meV by using equation (15) and the maximum value of 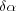 is obtained as 0.65′′ by using equation (14). By differentiating equation (15), we have the energy resolution d*E* obtained from the position of the mirage interference fringes with the position resolution d*x* as
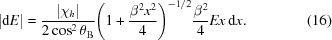
When d*x* is 0.1 mm and 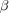 = 0.63 mm^−1^ in the case of Si(220), 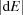 is approximately 0.4 meV at *x* = 1.5 mm. This means that the mirage interference fringes from the third crystal can be used for energy analysis of the beams with an energy resolution of sub-meV. The value of 0.1 mm for d*x* is nearly the same as the projected width of Slit 4 on the sample surface (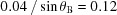 mm).

In the case of angle dispersive diffractometry, an asymmetric reflection is usually used as a monochromator or an analyzer with high energy resolution from meV to sub-meV. For example, two asymmetrically cut crystals with (+*n*, +*m*, −*m*, −*n*) setting were used as a monochromator with the energy bandwidth of meV (Ishikawa *et al.*, 1992 ▶; Yabashi & Ishikawa, 2000 ▶). According to the relation 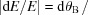 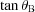, it is also possible to have X-rays with a small energy bandwidth by using back reflection. X-rays with an energy resolution of 0.45 meV were obtained by using Si(13 13 13) reflection with 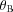 = 89.98° for X-rays of 25.70 keV (Verbeni *et al.*, 1996 ▶). Another monochromator with an energy resolution of sub-meV was designed by combining asymmetric reflection with back reflection (Baron *et al.*, 2001 ▶; Stoupin *et al.*, 2013 ▶). Stoupin *et al.* designed a monochromator with high spectral efficiency by combining asymmetric reflection from an asymmetrically cut diamond with back reflection from a silicon crystal. In these angle dispersive monochromators, it is necessary to choose an appropriate crystal and its Bragg reflection after setting the X-ray energy. In contrast, it is possible to adjust the energy resolution for any energy of X-rays just by choosing a Bragg reflection and changing the strain gradient parameter 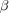 for the current monochromator using mirage interference fringes.

As the FWHM of the rocking curve of 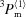 in Fig. 6 ▶ is 0.6′′, which is much smaller than that of 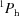 (9′′) in Fig. 5 ▶(*a*), the rocking curve of 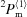 is regarded to be the intrinsic curve of the primary diffraction beam of 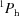. The value of 0.6′′ actually corresponds to the FWHM of 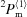. However, according to the estimation by using the beam width passing through Slit 2 with a width of 0.02 mm, the FWHM of the rocking curve of 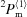 should be 0.2′′. The measured FWHM is three times larger than this estimated value. The cause of this difference is probably the source size (0.06 mm) of the synchrotron radiation X-rays from the bending magnet. If the source size is assumed to be 0.06 mm, the FWHM of the rocking curves of 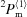 becomes 0.6′′, which agrees with the observed maximum value of 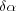 ≃ 0.65′′. If we use an undulator instead of the bending magnet, we can obtain a beam with a much smaller divergence angle by using this mirage diffractometer, because the source size of the undulator is approximately ten times smaller than that of the bending magnet.

**Figure 8:**
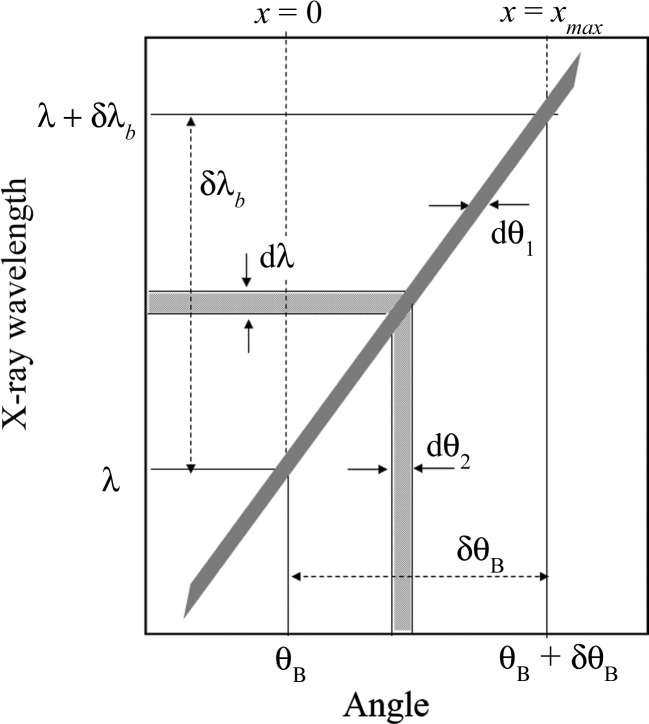
DuMond diagram. 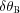 (= 0.07′′) is the angular width of the incident beam on the second crystal used as a monochromator through Slit 2, d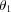 (= 0.003′′) that on the third crystal used as the sample through Slit 3 and d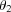 (= 0.003′′) the angular resolution of the analyzer obtained by moving Slit 4. 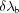 (= 1.1 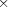 10^−7^ nm) is the bandwidth of the X-ray wavelength through Slit 2 and d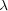 (= 4 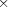 10^−9^ nm) the wavelength resolution of the analyzer obtained by moving Slit 4.

Fig. 8 ▶ shows a DuMond diagram to demonstrate the angle and energy resolutions of the current triple-crystal difftactometer with (+, −, +) setting. The reflection indices of 220 are the same for the three Si crystals. The glancing angle of the incident beam on each crystal is fixed when the mirage interference fringes are observed by moving Slit 4 in Fig. 3 ▶(*d*). The divergence of the Bragg angle 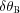 (= 0.07′′) and the bandwidth of the wavelength 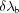 (= 1.1 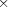 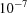
 nm) of the second crystal used as a monochromator are determined according to the source size of the synchrotron radiation X-rays as described above. The divergence angle d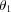 of the beam from the first peak of the mirage interference fringes in Fig. 4 ▶(*a*) is found to be 0.003′′ by using equations (4), (14) and (16), after passing Slit 3 of 40 µm width. The value of d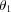 corresponds to the angular width of the 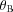 − 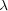 curve. As the mirage interference fringes from the third crystal are observed by moving Slit 4 with a vertical width of 40 µm, the angular width d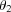 of the beam from Slit 4 is 0.003′′ and the bandwidth of the wavelength d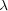 is 4 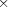 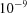
 nm, which is much smaller than 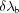, as shown in Fig. 8 ▶. Slit 4 is located at 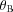 when *x* = 0 mm and at 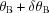 when *x* = 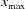. The energy width d*E* is approximately 0.4 meV by using the relation 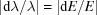.

## 5. Summary   

In the present experiment, the divergence angle of the beam from the first Si crystal is 4′′. When this beam is incident on the second Si crystal, the divergence angle of the mirage interference fringes is reduced to 0.6′′. The second crystal works as a monochromator to obtain a small divergence angle and thus high energy resolution. Table 1 ▶ shows the peak positions *x* and the corresponding values of 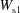 (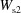), 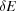 and 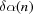 of mirage interference fringes from the third crystal. 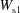 and 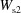 are the values of 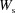 obtained by using equation (8) for the refracted beams 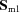 and 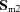 in Fig. 1 ▶(*b*). The intensity of the diffracted beam corresponding to 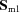 is approximately five times larger than that corresponding to 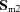, as shown in Fig. 7 ▶. This is because beam 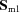 directly reaches *A*_2_, while beam 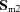 reaches point *A*_2_ after being once reflected from the top surface. Then we use the value of 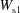 for estimating 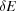 and 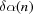. The average value of the difference between two adjacent values of 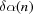 given by 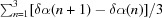 is 0.1′′, which agrees with the angular difference between two adjacent peaks of 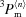 in Fig. 7 ▶. The value of energy width 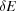 obtained in Table 1 ▶ shows that the third crystal works as an analyzer with high energy resolution.

In order to have X-rays with a very small divergence angle, an asymmetric crystal monochromator is widely used. When we use an Si(220) asymmetric monochromator for an X-ray energy of 11 100 eV, for example, the Bragg angle is 16.9° and the asymmetric factor *b* [= 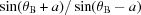 with *a* = 15.9°] is 31. The divergence angle from the asymmetric crystal monochromator is 1/5.6 (= 1/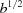) of the divergence angle from a symmetric crystal, and the X-ray intensity is reduced by 1/31. If we use the mirage interference fringes from Si(220) as a monochromator, on the other hand, the divergence angle is 1/8 of the incident X-ray and the intensity is reduced by 1/50, because the number of photons of the incident beams through the second crystal is approximately 25 000 s^−1^ and that of the mirage interference fringes from the third crystal is 500 s^−1^ according to the present experiment, as shown in Fig. 4 ▶. The intensity is the same as or slightly weaker than that from an asymmetric crystal. It is an advantage of this monochromator using the mirage interference fringes that the setup is quite easy and its structure is simple, although the divergence angle and the intensity are the same orders of magnitude as those of an angle dispersive monochromator using an asymmetric crystal.

Fukamachi *et al.* (2011 ▶) reported a monochromator with a very small divergence angle using the multiple-Bragg Laue mode diffraction from a lateral surface. The characteristics and usability of this instrument are nearly the same as those of the present monochromator using mirage interference fringes, but the divergence angle from the monochromator using the multiple-Bragg Laue mode is about twice that using mirage diffraction.

In this experiment, we have measured the intensities of mirage diffraction with a scintillation counter by moving a slit of very small width in front of it. The intensity distribution of the mirage interference fringes as a function of *x* is regarded as a spectrum of the incident X-rays projected onto the crystal according to equation (15). If we use an X-ray CCD camera or a position sensitive detector instead of a scintillation counter, the energy resolution of the spectrum should be improved to a large extent and the measuring time should be reduced. As we have very large angle amplification in the mirage diffraction, in the near future we can expect to achieve high energy resolution (less than meV) by using this diffractometer if it is combined with X-rays from an undulator beamline and a CCD camera.

## Figures and Tables

**Table 1:** The peak position *x* of mirage interference fringes of 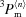 for *n* = 1, 2, 3 and 4, and the corresponding values of 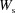, 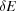 and 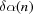

*n*	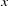 (mm)	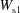 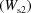	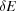 (meV)	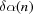 (′′)
1	1.41	−1.10 (−1.03)	−3.0	−0.18
2	1.96	−1.17 (−1.05)	−5.0	−0.30
3	2.27	−1.23 (−1.06)	−6.7	−0.40
4	2.51	−1.27 (−1.07)	−7.9	−0.48
