# An X-ray diffractometer using mirage diffraction. Erratum

**DOI:** 10.1107/S1600576714026028

**Published:** 2015-01-30

**Authors:** Tomoe Fukamachi, Sukswat Jongsukswat, Dongying Ju, Riichirou Negishi, Keiichi Hirano, Takaaki Kawamura

**Affiliations:** a Saitama Institute of Technology, Fukaya, Saitama 369-0293, Japan; b Institute of Material Structure Science, KEK-PF, High Energy Accelerator Research Organization, Tsukuba, Ibaraki 305-0801, Japan; c University of Yamanashi, Kofu 400-8510, Japan

**Keywords:** mirage diffraction, mirage fringes, interference fringes, X-ray difractometers, monochromators, dynamical theory of X-ray diffraction

## Abstract

Errors in the article by Fukamachi, Jongsukswat, Ju, Negishi, Hirano & Kawamura [*J. Appl. Cryst.* (2014), **47**, 1267–1272] are corrected.

In the article by Fukamachi *et al*. (2014 ▶), there are errors in Fig. 2 (§2, p. 1268) and in equations (12)–(16) (§4, p. 1270–1271).

The equations should read
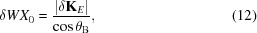


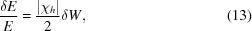


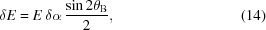


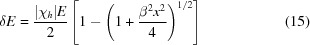
and 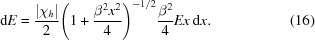
Then, rather than the value of 11 meV for |δ*E*| in the first line of the left column on p. 1271, the value should read 10 meV. Rather than −3.0, −5.0, −6.7 and −7.9 meV for the values of 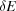 in Table 1, they should read −2.7, −4.6, −6.2 and −7.3 meV, respectively. In spite of these corrections, the conclusions are not affected.

As the last sentence of the caption of Fig. 2 of Fukamachi *et al*. (2014 ▶), the following sentence should be added: 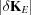 is the distance between *a*_2_ and *a*_4_. Fig. 2 of Fukamachi *et al*. (2014 ▶) is updated as Fig. 1 ▶ in the current article to reflect the correction.

## Figures and Tables

**Figure 1:**
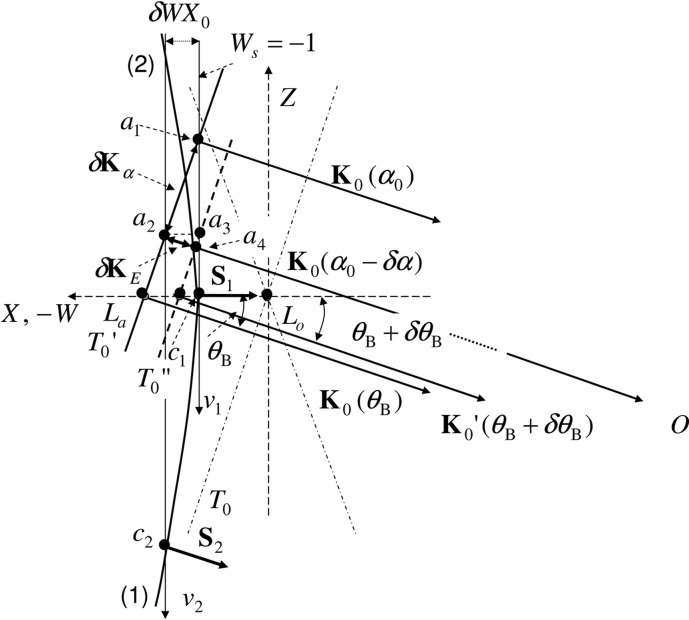
As the last sentence of the caption of Fig. 2 of Fukamachi *et al*. (2014 ▶), the following sentence should be added: 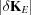 is the distance between *a*_2_ and *a*_4_.
